# Water Chemistry beneath Graphene: Condensation of a Dense OH–H_2_O Phase under Graphene

**DOI:** 10.1021/acs.jpcc.1c10289

**Published:** 2022-02-23

**Authors:** Elin Grånäs, Ulrike A. Schröder, Mohammad A. Arman, Mie Andersen, Timm Gerber, Karina Schulte, Jesper N. Andersen, Thomas Michely, Bjørk Hammer, Jan Knudsen

**Affiliations:** † Division of Synchrotron Radiation Research, Department of Physics, Lund University, Box 118, 221 00 Lund, Sweden; ‡ II. Physikalisches Institut, Universität zu Köln, 50937 Köln, Germany; ¶ Aarhus Institute of Advanced Studies, Aarhus University, Aarhus C, DK-8000 Denmark; § MAX IV Laboratory, Lund University, Box 118, 221 00 Lund, Sweden; ∥ Deutsches Elektronen-Synchrotron (DESY), 22607 Hamburg, Germany; ⊥ Department of Physics and Astronomy - Center for Interstellar Catalysis, Aarhus University, Aarhus C, DK-8000 Denmark; # Division of Synchrotron Radiation Research, Department of Physics, Lund University, Box 118, 221 00 Lund, Sweden

## Abstract

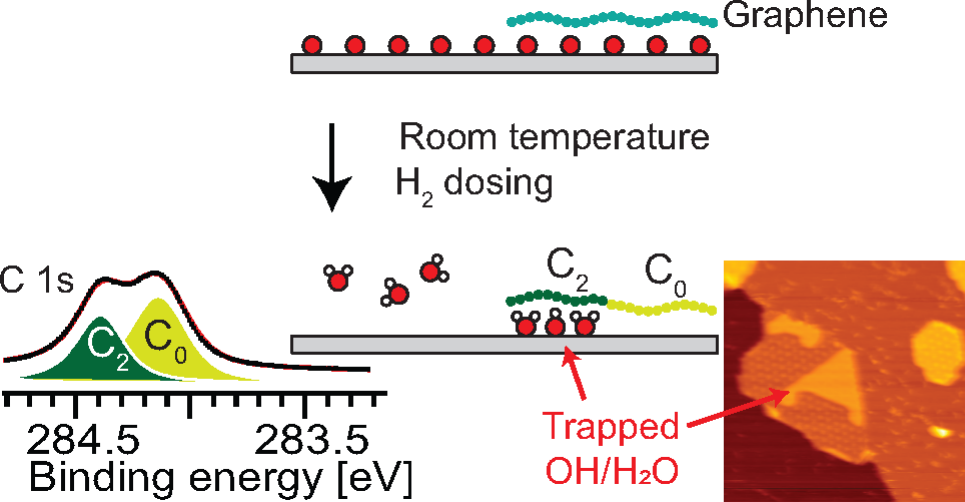

Room temperature oxygen hydrogenation below graphene flakes supported by Ir(111) is investigated through a combination of X-ray photoelectron spectroscopy, scanning tunneling microscopy, and density functional theory calculations using an evolutionary search algorithm. We demonstrate how the graphene cover and its doping level can be used to trap and characterize dense mixed O–OH–H_2_O phases that otherwise would not exist. Our study of these graphene-stabilized phases and their response to oxygen or hydrogen exposure reveals that additional oxygen can be dissolved into them at room temperature creating mixed O–OH–H_2_O phases with an increased areal coverage underneath graphene. In contrast, additional hydrogen exposure converts the mixed O–OH–H_2_O phases back to pure OH–H_2_O with a reduced areal coverage underneath graphene.

## Introduction

One promising application of the rich variety of new 2D materials is as model systems for studying catalysis. For example, single atom doping of 2D materials can be used to create new catalytic sites, single atom or substrate doping can used to alter the catalytic activity of the 2D material itself, the 2D material can be used like a chain armor to protect catalyst materials against harsh reaction conditions, the 2D material can be used as membrane that only allows for example protons to penetrate while more bulky ions or molecules are blocked,^1^ or the confined space between the 2D material and its metal substrate can be used to create new reaction pathways.^2^ Examples of this last application were reviewed extensively in 2017 by Fu and Bao covering elemental and molecular intercalation both from gas and liquid phase as well as undercover growth and reactions.^3^ Since then an impressive amount of literature has been published within the area of “undercover catalysis”, and it has for example recently been demonstrated that defective graphene on Pt(111) can increase rates for the hydrogen evolution reaction by 200% as compared to bare Pt(111).^4^

Many intercalation studies of atoms and molecules under graphene have been published in the past and they have given an excellent starting point for understanding undercover reactions. For example, oxygen, CO, and hydrogen intercalation has been studied in detail for graphene (Gr) grown on a number of metal surfaces including Ru(0001),^5−13^ Pt(111),^13,14^ and Ir(111)^5,15−20^ with experimental surface science techniques as well as density functional theory. These studies have given unprecedented understanding of intercalation of molecules under Gr, the intercalation mechanisms, the phases formed under the Gr cover and their experimental fingerprints. Also, it has been shown that Gr intercalation can be controlled by gas pressure, temperature, and the morphology of the epitaxial Gr on its substrate.

In contrast to the rich amount of intercalation studies under Gr fewer studies report on undercover gas-phase reactions. The best studied system is CO oxidation under Gr grown on Pt(111). Low electron energy microscopy (LEEM) studies^14,21^ performed at low pressures in the group of Bao revealed that wrinkles in the graphene layer function as one-dimensional gas inlets for oxygen and CO. A modified CO adsorption structure and a lowered CO desorption temperature result from the Gr layer above. Furthermore, it was demonstrated how in situ LEEM can be used to follow CO oxidation of rows of CO molecules trapped below Gr close to the Pt step edges. More recently^22^ the same group used a combination of polarization-modulated infrared reflection absorption spectroscopy (PM-IRRAS) and ambient pressure XPS (APXPS) to study CO oxidation below the submonolayer and one monolayer Gr on Pt(111) in a pressure range from 10^–9^ to 40 mbar. Interestingly, these experiments revealed that the Gr overlayer weakens the CO-Pt interaction and thereby lowers the activation energy for CO oxidation. The authors even demonstrated that it is possible to run the CO oxidation reaction over a Pt(111) surface fully covered by Gr. CO oxidation below graphitic shells has also been demonstrated for Pt nanoparticles where a lower activation energy was demonstrated.^23^

Some of the authors of this paper investigated CO oxidation over an oxygen saturated Ir(111) surface below Gr flakes at elevated temperatures, using in situ XPS at low pressures (5 × 10^–9^ mbar) of CO and elevated temperatures (490 K).^15^ At these conditions, our study showed that the oxygen atoms below the Gr flakes work as a reservoir from which oxygen is expelled. Consequently, CO oxidation only takes place at the bare Ir(111) patches.

For hydrogen oxidation using Gr flakes supported by Pt-group metals, we are only aware of some evidence for formation of H_2_O and/or OH on Pt(111) under Gr upon subsequent oxygen and hydrogen dosing based on O 1s and C 1s XP spectra.^3^ However, no detailed analysis or discussion was presented. In contrast, hydrogen oxidation below free-standing SiO_2_ bilayer film supported by Ru(0001) was recently studied in detail by Prieto et al.^24^ In this study, it was demonstrated that the apparent activation energy determined from a front velocity analysis of LEEM images is reduced by a factor of 2 for the confined reaction.

Here, we report on a detailed study of H_2_ oxidation on Ir(111) with and without Gr. At room temperature and without Gr, water forms and desorbs instantaneously, while the identical reaction performed under the Gr cover leads to trapping of a dense mixed OH–H_2_O structure. Furthermore, we demonstrate how the Gr doping level together with C 1s reference values for intercalated structures^19^ give a novel tool to follow undercover reactions in situ. Using this new tool we follow how the dense OH–H_2_O structure increases its areal cover underneath Gr once O atoms are dissolved into it and subsequently reduce its areal coverage upon converting the dissolved O atoms to OH and H_2_O once H atoms are dissolved into the structure.

## Experimental Details

XPS experiments were collected in normal emission with an angular acceptance of ±5° at the now closed beamline I311^25^ at the MAX IV Laboratory. Photon energies of 120 eV for Ir 4f, 390 eV for C 1s, and 625 eV for O 1s were used. Reproducible C 1s core level shifts (CLS) as small as 20 meV can be measured on this beamline making it ideally suited for characterizing the sharp and intense C 1s peak of graphene. STM and TPD measurements were carried out at the TUMA-III Laboratory in Cologne. All STM imaging was conducted at room temperature and the STM topographs were postprocessed in the WxSM software.^26^ The base pressures of the XPS and STM setups were below 1 × 10^–10^ mbar. Details of the TPD experiments are explained in the Supporting Information when discussing Figure S3.

Gr was grown on a Ir(111) single crystal with the same recipe as described in previous publications.^15^ Oxygen exposure onto Gr is conducted in an O_2_ pressure of 5 × 10^–6^ mbar (200 L) if not specified otherwise. The dose of 200 L is more than an order of magnitude larger than what is necessary to reach saturation coverage of oxygen on Ir(111)^27,28^ and full intercalation of 0.5 ML Gr on Ir(111). H_2_ exposure is conducted at 5 × 10^–7^ mbar (100 L), if not specified otherwise. No further change in the C 1s spectrum was seen at higher hydrogen doses.

## Calculational Details

Density functional theory calculations were performed using an evolutionary search algorithm^29,30^ to determine the structure and stability for OH–H_2_O mixed phases intercalated under an idealized (4 × 4) graphene covered  Ir(111) surface unit cell. A five layer Ir slab was used with four of the layers being fully relaxed. The calculations were performed with the real-space projector augmented wave GPAW code^31^ using the “dispersion-aware” M06-L functional^32^ which has proven successful in describing, e.g., the bonding in layered compounds^33^ and the hydrogenation of graphene over Ir(111).^34^ Throughout, the graphene lattice constant of 2.45 Å was used together with (2 × 2) **k**-points in the surface Brillouin zone and a grid-spacing of 0.175 Å. For calculating the core level shifts (CLSs), the fully screened core hole approximation was used, meaning that the self-consistent total energy of the system including the core hole was evaluated, thus including final state effects. The reported shifts are referenced to a C atom in nonintercalated graphene on Ir. In practice this was done by calculating the shift in each system (nonintercalated or intercalated graphene) with respect to a single C atom attached to the bottom side of the Ir slab. Subtraction of these shifts then yields the relative shift of each system with respect to the nonintercalated reference. With the used sign convention, a positive CLS corresponds to a shift to higher binding energies in the experiment.

## Results and Discussion

**Figure 1:**
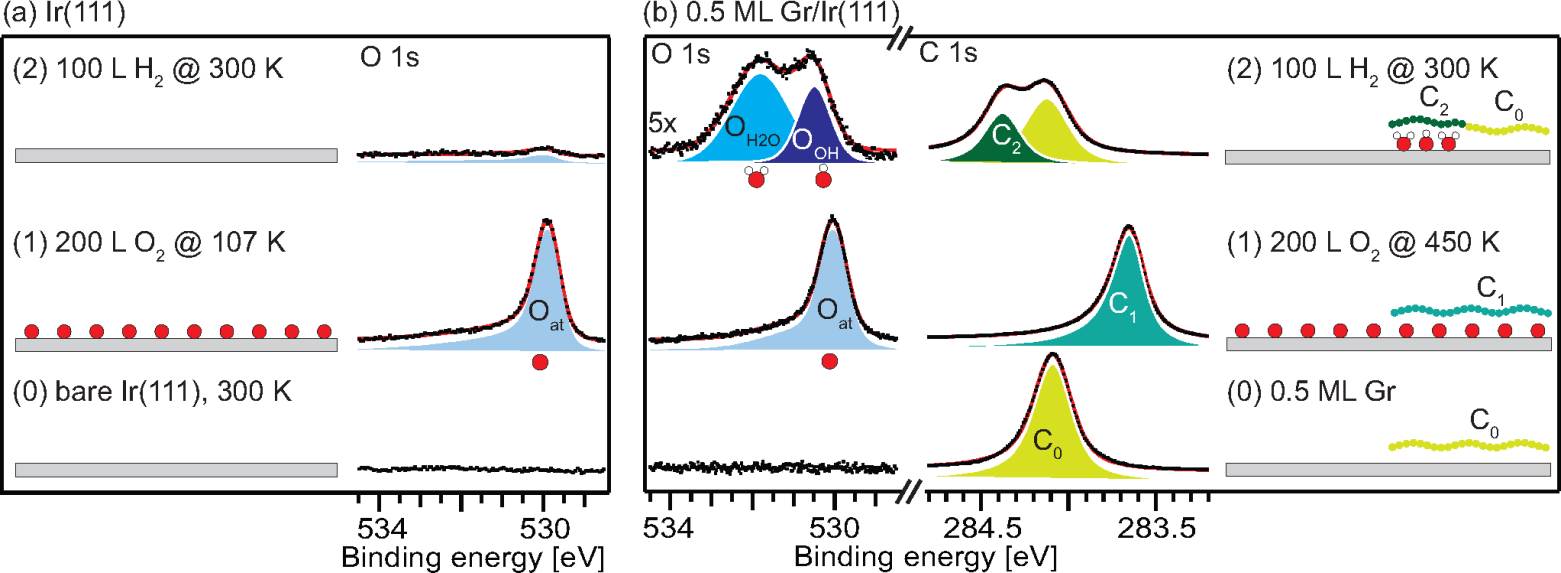
Comparison of XPS spectra after exposing Ir(111) subsequently to oxygen and hydrogen with and without graphene. (a) O 1s spectra from (0) clean Ir(111), (1) after exposure to 200 L O_2_ at 107 K, and (2) after subsequent exposure to 100 L H_2_ at 300 K. (b) O 1s and C 1s spectra from (0) pristine 0.5 ML Gr, (1) after exposure to 200 L O_2_ at 450 K, and (2) after subsequent exposure to 100 L H_2_ at 300 K. All experimental details and additional spectra are given in the Supporting Information (see Figure S1).

Before H_2_ oxidation under Gr is discussed, we briefly describe the oxidation without Gr on Ir(111). Figure 1a shows from bottom to top the O 1s spectra of Ir(111): (0) after cleaning, (1) after oxygen exposure until saturation which results in a *p*(2 × 1)-O structure with O atoms adsorbed in the 3-fold hollow sites,^15,27^ and (2) after subsequent hydrogen exposure reacting the adsorbed oxygen to water, that instantaneously desorbs. Due to the rapid desorption of the product water at the reaction temperature of 300 K, atomic oxygen (with a binding energy of 530.0 ± 0.05 eV for the O 1s core level) is the only oxygen containing species we observed. Oxygen was in these experiments initially dosed at 107 K to test for water formation and trapping at temperatures of 107 and 170 K, respectively, caused by 100 L H_2_ exposure. In contrast to the final H_2_ exposure at 300 K, these experiments left the oxygen phase untouched.

The process is dramatically different when Ir(111) is half covered by Gr flakes (0.5 ML), as can be seen in panel b. Prior to hydrogen dosing, the sample was saturated with oxygen by dosing 200 L of O_2_ at 450 K. A slightly elevated sample temperature is needed to facilitate oxygen intercalation under the Gr flakes. As established in our previous work,^15^ the mobile O atoms resulting from dissociative chemisorption are pushed under the Gr flakes until these are completely delaminated and a *p*(2 × 1)-O structure forms on the entire sample, both on the bare Ir(111) and under the flakes. As the O atoms bind downward to Ir(111) the O 1s binding energy position is the same for oxygen atoms with and without the Gr cover. However, the delamination of the Gr flakes is clearly signaled by a C 1s peak shifted −0.47 eV compared to pristine graphene, here called C_1_, in agreement with our previous work and the work of Laciprete et al.^15,16^ Upon H_2_ exposure, the molecular hydrogen adsorbs dissociatively on the areas not covered by Gr.^18^ The H atoms can now follow two different reaction paths in which they titrate away atomic O. They either (i) react with O on Ir(111) patches not covered by Gr or they (ii) react with O atoms under Gr. For reaction path i, we already demonstrated that water will form and desorb directly. Reaction path ii would lead to OH and/or H_2_O molecules being formed, and possibly trapped, under Gr.

Comparing the spectra in panel b, before (1) and after (2) H_2_ exposure, it is obvious that the O_at_ and C_1_ components disappear, giving evidence that the *p*(2 × 1)-O structure is fully removed. Furthermore, new oxygen components develop at 530.4 ± 0.1 eV, O_OH_, and 531.9 ± 0.1 eV, O_H2O_, respectively, simultaneously with the reappearance of the C_0_ component (59%) signaling nonintercalated graphene, and a new component C_2_ at 284.37 eV (41%). Shavorskiy et al.^35^ previously studied low temperature water adsorption and desorption on oxygen covered Ir(111) with high resolution XPS and assigned similar O 1s components observed at 530.5 and 531.5 eV to a mixed phase of OH and H_2_O on Ir(111). Based on the simultaneous appearance of the new C_2_ component and the OH and H_2_O components of the mixed phase, we conclude that trapped OH and H_2_O formed under 41% of the graphene flakes via reaction path ii and caused the new C_2_ component. Evidence for H atoms following reaction path i comes from the finding that H_2_ dosing leads to a reduction of the O 1s intensity to 35 ± 10% of the initial coverage of the *p*(2 × 1)-O structure.

The local coverage of the mixed OH–H_2_O phase causing the C_2_ component can be found from the absolute intensities of the O_OH_ and O_H2O_ components and the relative intensity of the C_2_ component. To obtain a precise value for the coverage of the mixed OH–H_2_O phase we dosed hydrogen onto O-intercalated Gr flakes without moving the sample and while measuring the C 1s and O 1s regions simultaneously (see Figure S2). From this experiment we find that the mixed OH–H_2_O phase has a surprisingly high local density of 0.8 O-containing molecules per Ir surface atom. This density is almost the double of the experimentally determined O-density for the *p*(2 × 1)-O structure (0.44 O atoms per Ir surface atom). The high density implies that O atoms trapped under Gr condense into a dense mixed OH–H_2_O phase upon reaction with hydrogen. The reappearance of the C_0_ component in panel 2 of Figure 1b is a necessary consequence of the higher oxygen density of the dense OH–H_2_O phase.

Further support for the formation of OH and H_2_O under Gr comes from a temperature-programmed desorption (TPD) experiment in which 0.5 ML Gr on Ir(111) was exposed sequentially to O_2_ and H_2_. The H_2_O desorption peak temperature of 440 K (see Figure S3) is significantly higher than the desorption peak temperature of the pure H_2_O bilayer (170 K)^35−37^ or a mixed OH–H_2_O phase (210–235 K) formed on bare Ir(111) by dosing water onto an O precovered Ir(111) surface at low temperatures.^35,36^ Possible reasons for the much increased desorption temperature for the phase formed under Gr are discussed below.

**Figure 2:**
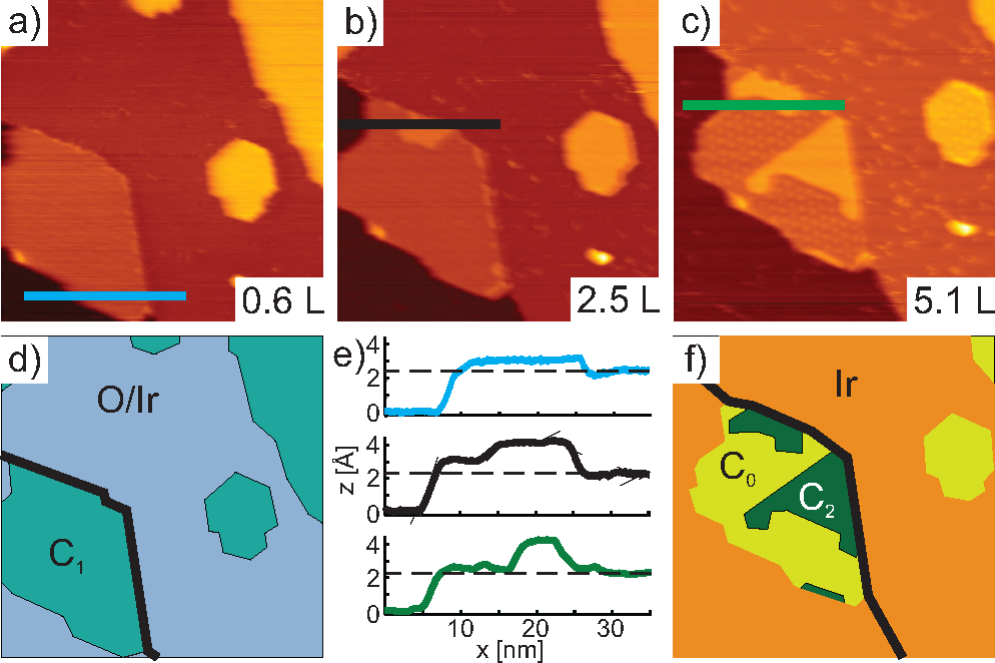
(a–c) Snapshots (71 × 71 nm^2^) from an STM movie showing three O-intercalated Gr flakes while they are exposed to 5 × 10^–9^ mbar of H_2_ (see Supporting Information for full movie). (d and f) Schematic representations of panels a and c, respectively. The colors used are in line with those used for the XPS components and the black lines show Ir step edges. (e) Line scans along the lines marked in panels a–c.

The formation of the dense OH–H_2_O phase under oxygen-intercalated Gr can be followed in real time by acquiring scanning tunneling microscopy (STM) movies during H_2_ exposure. Panels a−c of Figure 2 show snapshots from such a movie taken during exposure to 5 × 10^–9^ mbar of H_2_ at room temperature (the full movie can be seen in the Supporting Information), while panels d and f of Figure 2 show schematic representations of panel a and c, respectively. Upon H_2_ exposure the formation of OH–H_2_O islands beneath Gr is visible as bright areas. After the H_2_ intercalation has terminated Gr appears in two heights as evident by the linescans in Figure 2e. The lower areas arise from Gr that is no longer intercalated and thus is laminated to the Ir substrate, while the 1.5 Å higher areas arise from the dense OH–H_2_O phase (see also Figure 2f). The phase boundary between the OH–H_2_O phase and the nonintercalated Gr is sharp and follows the moiré pattern as visible in Figure 2c. Apparently, the chemical inhomogeneity of Gr’s binding to Ir(111) affects the shape of the OH–H_2_O phase formed beneath.

Based on our XPS, TPD, and STM results, we conclude that room temperature hydrogen dosing onto O-intercalated Gr flakes leads to a mixed dense OH–H_2_O phase exclusively formed and trapped under Gr. The intercalated OH–H_2_O phase remains trapped under Gr up to a temperature of 440 K. In contrast, upon room temperature H_2_ exposure of oxygen adsorbed to bare Ir(111) the reaction products desorb instantaneously.

**Figure 3:**
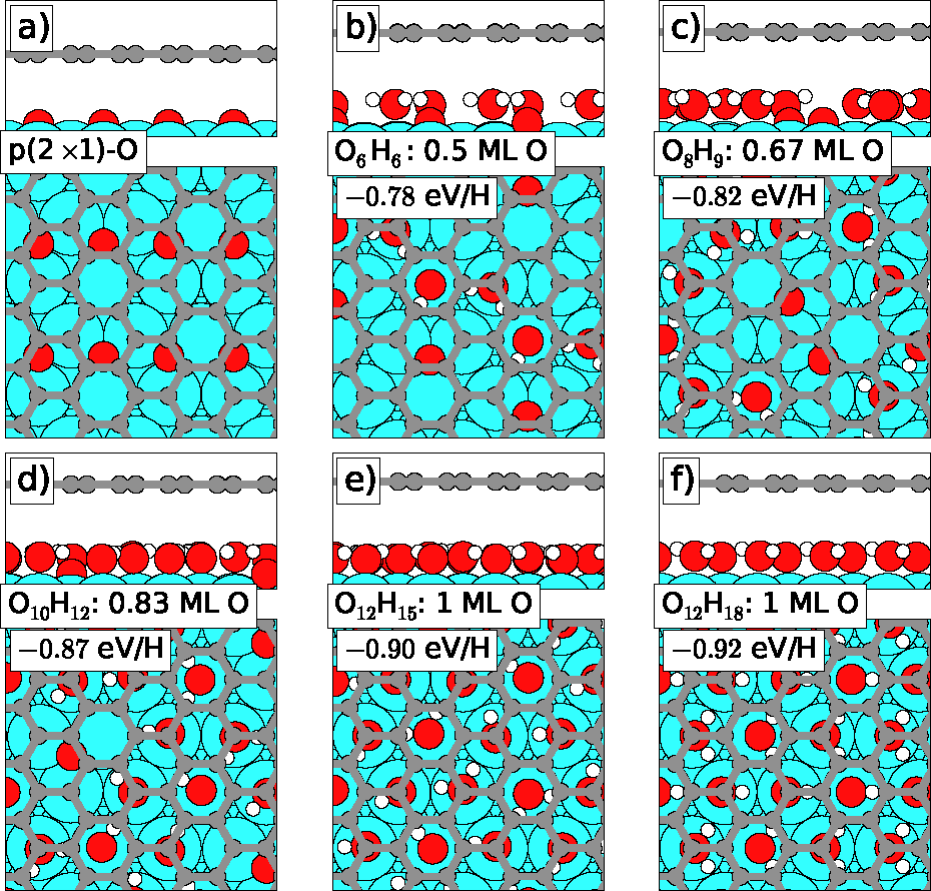
DFT-calculated structures identified with an evolutionary search algorithm upon H-uptake in the *p*(2 × 1)-O structure. The adsorption energy per H atom in eV upon adsorption from the gas phase and the density (as measured by the O coverage) are included for each structure. In the O_*x*_H_*y*_ labels, *x* and *y* indicate the number of O and H per  Ir(111) unit cell.

Our finding of an intercalated dense OH–H_2_O mixed phase is supported by the density functional theory calculations summarized in Figure 3. The stoichiometry and density of the mixed phase was varied from the O density of the *p*(2 × 1) phase (0.5 ML) (Figure 3a) and a 1:1 H:O ratio (Figure 3b) up to twice that O density (1 ML) and a 3:2 H:O ratio (Figure 3f). Assuming the mixed phases to result from H_2_ exposure, the denser phases imply various degrees of contraction of the intercalated structures, i.e., up to 50% contraction for the densest structures shown in Figure 3, parts e and f. In Figure 3 is also shown the calculated average adsorption potential energies of the H atoms relative to H in the hydrogen molecule. These calculations reveal that the energy gain per H atom increases from 0.78 eV to more than 0.9 eV as the structures develop from a 1:1 H:O ratio and no contraction to denser and more H-rich structures. We note that by using the evolutionary search algorithm for the structural search (covering several hundred different structural candidates per stoichiometry and density) our computed structures are unbiased by any expectations or experimental knowledge. We thus attribute significance to the finding at mid-densities—Figure 3c,d—of atomic O species dissolved in the OH–H_2_O network and to the finding of alternating OH and H_2_O rows in the most dense structure, Figure 3f. For this O_12_H_18_ phase (formally a *p*(2 × 1)–OH–H_2_O phase), we find a DFT calculated core level shift (CLS) of +0.18 to +0.22 eV (the CLS is slightly different for the different C atoms in graphene) agreeing relatively well with the experimentally observed shift of +0.28 eV for the C_2_ component. Furthermore, the finding in DFT of a preference for highly dense phases fits well with the experimental observations of a local coverage of the OH–H_2_O structure (0.8 ML), which is approximately double that of the *p*(2 × 1)-O structure (0.44 ML), as well as with the partial relamination of the Gr flakes that occurs in the oxygen-intercalated regions once the oxygen leaves these regions upon forming the dense phases.

At this point, it should be underlined that we did not perform a complete structural characterization of the intercalated OH–H_2_O phase here. This would require structural information on the intercalated phase, which we did not observe in our STM imaging. Most plausible, the OH–H_2_O dense phase below graphene flakes is at room temperature not perfectly ordered and displays slight local density variations. Nevertheless, the thermodynamic driving force for the formation of dense OH–H_2_O phases below graphene found in DFT explains the formation of the dense OH–H_2_O phase observed experimentally.

Comparing the DFT-calculated energies of the dense O_12_H_18_ phase with and without Gr, we determine an intercalation cost of 0.14 eV per OH–H_2_O unit. Thus, from the adsorption enthalpies alone we also expect preferential formation of the dense OH–H_2_O structure on bare Ir(111) patches in apparent contradiction to our experimental observations.

To explain this apparent contradiction, we studied the stability of various phases adsorbed on bare Ir(111) with respect to desorption of a water molecule. We assume that the barrier for water desorption can be described by the DFT-calculated desorption energy alone; i.e., we neglect any additional kinetic barriers. This should be a good approximation since water adsorption is typically a nonactivated process. Desorption of a water molecule from the dense O_12_H_18_ structure costs 1.36 eV, which according to the Redhead formula would correspond to a desorption temperature of 462 K and a residence time of 2.4 · 10^8^ s at 300 K if first order desorption, a frequency factor of 2.9 × 10^14^ s^–1^,^37^ and a heating rate of 5 K/s are assumed.

The dense O_12_H_18_ phase is therefore clearly stable on bare Ir(111) at room temperature. For comparison, we also considered the H-poor O_6_H_2_ phase as a model for the very first phases that would form upon the initial exposure of the *p*(2 × 1)-O structure to H_2_. The most stable structure found (see Figure S5 in the Supporting Information) consists of a single water molecule surrounded by atomic O. Desorption of this water molecule now costs only 0.77 eV, giving rise to a desorption temperature of 266 K and a residence time of 30 ms at 300 K. This phase is therefore clearly unstable with respect to water desorption at room temperature.

From these results, it is now evident why the dense O_12_H_18_ does not form at room temperature on bare Ir(111): As hydrogen is dosed onto *p*(2 × 1)-O without Gr, less dense phases containing water molecules surrounded by atomic O initially form. Since in these phases there is no possibility for attractive hydrogen bonding to neighboring OH groups or other water molecules (in contrast to the case of the denser phases shown in Figure 3), the O-surrounded water molecules are highly unstable and immediately desorb at 300 K. Thus, upon H_2_ exposure of the *p*(2 × 1)-O structure, the oxygen is simply titrated away, and the denser phases never have a chance to form, in perfect agreement with our experiments. Beneath Gr, the O_6_H_2_ phase also forms initially, but since it is impossible for water to desorb through the Gr film, the phase will be trapped. Continued hydrogen dosing and contraction will lead to the formation of the denser and highly stable phases such as O_12_H_18_.

Comparing the DFT-calculated desorption temperature of water from O_12_H_18_ on bare Ir (462 K) with the experimental desorption temperature of water from O_12_H_18_ trapped beneath Gr (440 K) we find a very good agreement. We note that the above-mentioned previous studies of water desorption from a mixed OH–H_2_O phase formed on bare Ir(111) found much lower desorption temperatures of around 210–235 K,^35,36^ but an explanation is hampered by the fact that no detailed characterization of the formed phases were given. A likely explanation for the lower desorption temperature in these previous studies is that H_2_O was dosed onto O-precovered Ir(111) while here the OH–H_2_O phase was formed by dosing H_2_ onto O-precovered Ir(111) with Gr islands. As OH can be formed without consuming surface oxygen if H_2_O is dosed onto O–Ir(111) the structures formed upon water dosing are expected to host also atomic oxygen.^35^ As discussed below this will lower the desorption temperature.

We have now found that one cycle of O intercalation and subsequent H_2_ exposure leads to formation of a dense OH–H_2_O structure exclusively intercalated under Gr. Next we will demonstrate that it is possible to dissolve oxygen into this structure, already at room temperature, leading to a less dense mixed structure containing: atomic oxygen, H_2_O, and OH. Furthermore, we will demonstrate how the C 1s signal of graphene can be used to probe the undercover reaction and, in particular, how this can be used to distinguish mixed and coexisting phases very easily.

**Figure 4:**
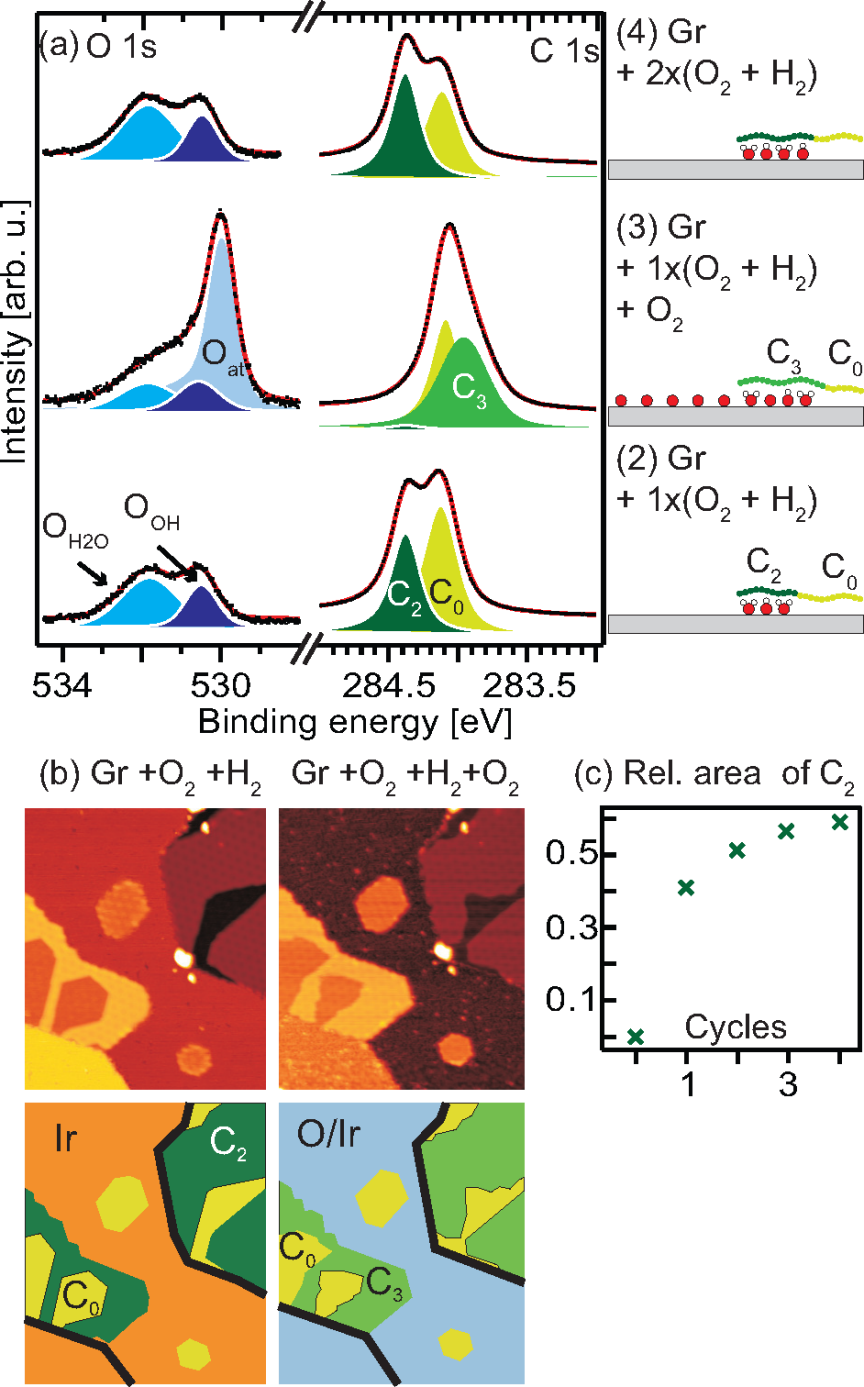
O-uptake in the superdense OH–H_2_O phase studied with XPS and STM. (a) O 1s and C 1s spectra of Gr exposed to (2) one cycle of (O_2_ + H_2_), (3) after an additional O_2_ exposure, and (4) after two cycles of (O_2_ + H_2_). (b) Left STM image: UHV, after 4 cycles of (O_2_+H_2_). Right STM image: During additional exposure to 1 × 10^–7^mbar of O_2_ after approximately 30 min (265 L). Image size 60 × 70 nm, *U* = −1 V, and *I* = 1 nA. Schematic representation of the STM images are shown below. The colors used are in line with those used for the XPS components and the black lines show Ir step edges. (c) Relative area of the C_2_ component versus the number of (O_2_ + H_2_) cycles obtained from XPS experiments.

In Figure 4a, we compare O 1s and C 1s spectra of Gr partly intercalated by the dense OH–H_2_O structure before and after an additional room temperature oxygen exposure cycle. Focusing first on the C 1s spectra it is clear that the C_2_ component assigned to the dense OH–H_2_O structure is removed upon the oxygen dosing and replaced by a broad C_3_ component located at 283.95 eV and thus shifted −0.14 eV, with respect to pristine graphene. No C_1_ component shifted by −0.47 eV and signaling the *p*(2 × 1)-O phase intercalated under Gr are observed. Therefore, formation of this phase is exclude. In contrast to the significant changes in the C 1s spectra, the OH and H_2_O components in the O 1s spectra are essentially unaffected by the oxygen dosing as demonstrated by Figure 4a and in additional in situ experiments shown in Figure S2. The simplest scenario required to reconcile these observations is that O dissolves into the OH–H_2_O structure, thereby modifying the OH–H_2_O interaction (turning C_2_ into C_3_), while keeping the amount of OH–H_2_O unchanged. Further, Figure 4a nicely demonstrates how the Gr doping level can be used to follow the reaction under cover. The disappearance of the C_2_ and the appearance of the C_3_ component are, for example, crucial for concluding that oxygen dissolves into the OH–H_2_O structure.

The in situ experiments in Figure S2 further reveal that the O_*at*_-coverage is reduced to half of the coverage of the *p*(2 × 1)-O structure, suggesting that the majority of the oxygen adsorbs on bare Ir(111) patches and only a small fraction is dissolved into the OH–H_2_O structure. Since the dense OH–H_2_O structure intercalated under Gr initially covers all Ir sites, we expect an intercalated O–H_2_O–OH structure to increase its coverage underneath the Gr cover causing more intercalation and less nonintercalated Gr. Both our XPS and STM data agree with this picture. In the XPS experiments we observe that the area of the C_0_ component is reduced by 23% when the dense OH–H_2_O structure is saturated with oxygen at room temperature (compare points 2 and 3 in Figure 4a), and inspection of the STM images in Figure 4b shows that the C_0_ marked areas are reduced by 34% (upper right corner) and 20% (lower left corner), respectively.

When the intercalated O–H_2_O–OH structure is exposed to H_2_ again we find that the dissolved oxygen can be fully converted to OH and H_2_O causing the C_2_ component to reappear with increased intensity. In more detail we observe that the C_3_ component, signaling the mixed O–OH–H_2_O phase, disappears upon H_2_ dosing while the C_2_ component, signaling the dense OH–H_2_O phase, reappears with a 10% increase in relative intensity (Figure 4, parts a and c). Upon continued cycles of subsequent O_2_ and H_2_ dosing at room temperature the relative intensity of the C_2_ component increases more and more for each cycle as shown in Figure 4c. The increase for each cycle is moderate, in agreement with our previous conclusion that only a smaller amount of oxygen atoms are dissolved into the OH–H_2_O phase upon room temperature oxygen dosing.

**Figure 5:**
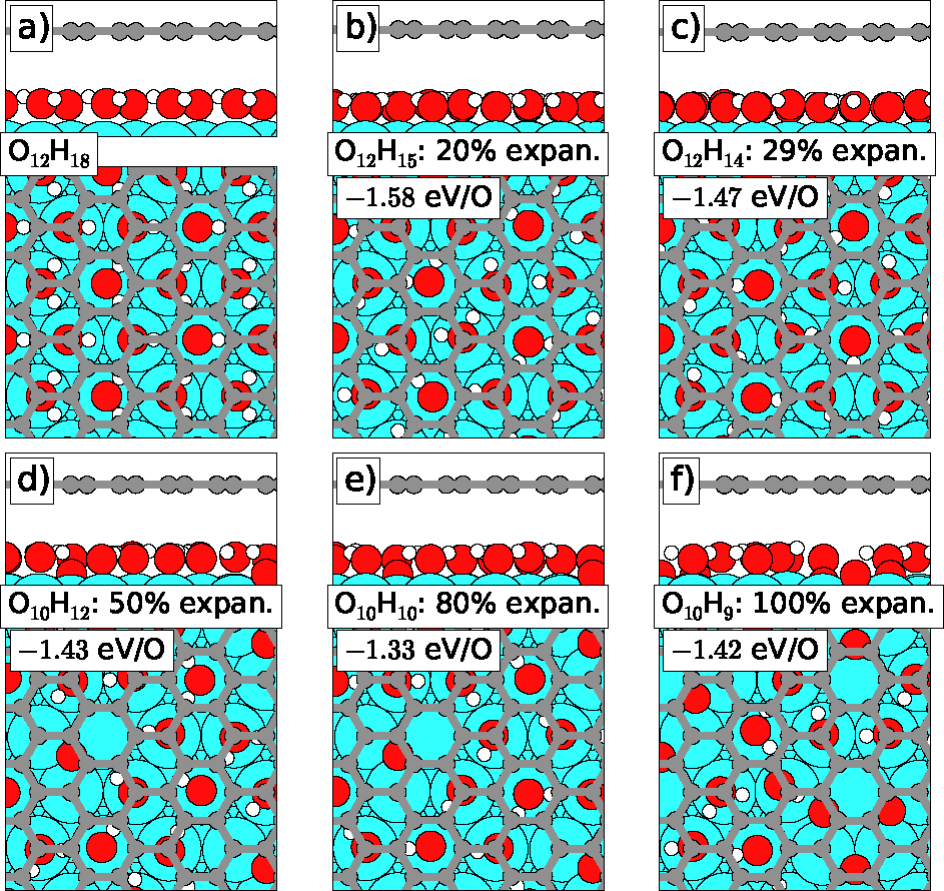
DFT-calculated structures revealed by an evolutionary search algorithm upon O uptake in the superdense OH–H_2_O phase. The adsorption energy per O atom in eV upon adsorption from the gas phase along with the degree of expansion of the structure with respect to the superdense OH–H_2_O phase are included in the figure.

The formation of the less dense O–H_2_O–OH structures under Gr upon O uptake in the dense OH–H_2_O phase is supported by our DFT calculations and the evolutionary search algorithm. Analogously to the calculations of the H uptake, the stoichiometry and density of O–H_2_O–OH structures were varied from the density of the O_12_H_18_ phase with a 3:2 H:O ratio down to half of that H density and a 9:10 H:O ratio. Our calculations, shown in Figure 5 together with the average adsorption potential energies of the O atoms given relative to O in the oxygen molecule, reveal that 1.6 to 1.3 eV per O atom is gained when oxygen dissolves in the dense OH–H_2_O phase and converts it to a more open structure. The best agreement between the measured C_3_ component and the DFT calculated CLS is found for the O_10_H_12_ and O_10_H_9_ phases. For these phases we find DFT calculated CLSs of −0.09 ± 0.01 (O_10_H_12_) and −0.17 ± 0.01 O_10_H_9_ close to the experimental position of the C_3_ component at −0.14 eV. The observed reduction of the nonintercalated Gr area of 20–34% in our STM and XPS experiments translate into a 29–49% increase of the intercalated area fitting best with the expected 50% increase of the areal coverage when the O_10_H_12_ structure is formed upon O-uptake.

## Conclusions

We investigated Ir(111) fully covered with chemisorbed oxygen and partly covered with Gr flakes where the oxygen is intercalated. Upon room temperature hydrogen exposure, adsorbed oxygen is titrated away from the bare Ir(111), while underneath graphene oxygen is hydrogenated giving rise to a dense OH–H_2_O phase. Ab initio calculations show that this dense phase is thermodynamically more stable than dilute OH–H_2_O phases. Experimentally, the dense OH–H_2_O phase is not observed on bare Ir(111), as its formation requires going through the dilute OH–H_2_O phases that simply desorbs without the confining cover of the Gr. We have demonstrated that the dense OH–H_2_O phase facilitates oxygen intercalation under Gr already at room temperature and by using the Gr doping level as an additional probe we showed that O atoms are dissolved into the OH–H_2_O phase making it less dense and increasing its areal coverage. Subsequent hydrogen dosing leads to the formation of more OH and H_2_O, and cycles of O_2_ and H_2_ dosing can therefore be used as an effective method to increase the area of the OH–H_2_O phase below the graphene flakes. We expect that many other molecules can be dissolved into the dense OH–H_2_O phase trapped below graphene. This finding paves the way for future studies of reactions taking place in dense OH–H_2_O phases mimicking the first layer of a water film on metal surfaces in simple room temperature experiments.

## Supporting Information Available

STM movie corresponding to Figure 2 (MP4)
Ir 4f spectra corresponding to Figure 1, O 1s and C 1s spectra recorded *in situ* while exposing the sample to O_2_ and H_2_, temperature-programmed desorption of the OH–H_2_O formed under Gr, O 1s and C 1s spectra acquired as a function of cycles of subsequent oxygen and hydrogen dosing, model of the most stable structure of the H-poor O_6_H_2_ phase (PDF)
