# Supplementary material for: Water Chemistry beneath Graphene: Condensation of a Dense OH–H2O Phase under Graphene
Source: J Phys Chem C Nanomater Interfaces. 2022 Feb 23;126(9):4347–54. doi: 10.1021/acs.jpcc.1c10289 (PMC8919254; doi:10.1021/acs.jpcc.1c10289)
Supplement: Supplementary file 2 — jp1c10289_si_002.pdf [file jp1c10289_si_002.pdf]

Supplementary Information for: Water Chemistry beneath Graphene: Condensation of a Dense OH-H_2O Phase under Graphene

Elin Grånäs,[†,‖] Ulrike A. Schröder,[‡] Mohammad A. Arman,[†] Mie Andersen,[¶,⊥] Timm Gerber,[‡] Karina Schulte,[§] Jesper N. Andersen,[†,#] Thomas Michely,[‡] Bjørk Hammer,[¶,⊥] and Jan Knudsen[*,†,#]

†*Division of Synchrotron Radiation Research, Department of Physics, Lund University, 22100 Lund, Sweden*

‡*II. Physikalisches Institut, Universität zu Köln, 50937 Köln, Germany*

¶*Aarhus Institute of Advanced Studies, Aarhus University, Aarhus C, DK-8000 Denmark*

§*MAX IV Laboratory, Lund University, 22100 Lund, Sweden*

‖*Deutsches Elektronen-Synchrotron (DESY), 22607 Hamburg, Germany*

⊥*Department of Physics and Astronomy - Center for Interstellar Catalysis, Aarhus University, Aarhus C, DK-8000 Denmark*

#*MAX IV Laboratory, Lund University, 22100 Lund, Sweden*

E-mail: jan.knudsen@sljus.lu.se

Supplement to figure Fig. 1

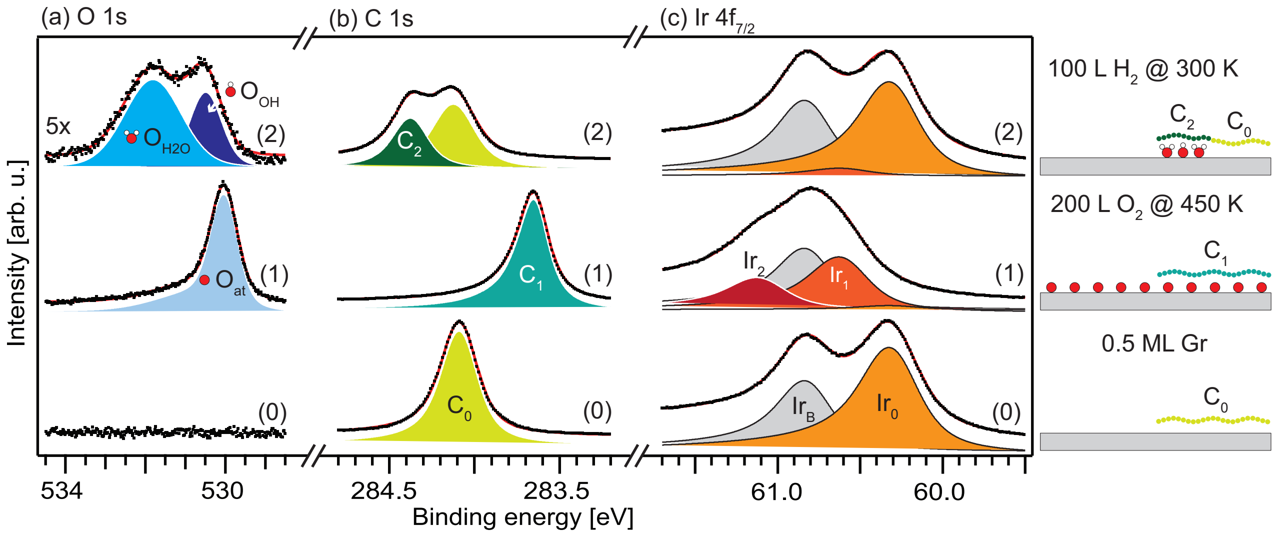

Figure S1: (a) O 1s, (b) C 1s, and (c) Ir 4f spectra corresponding to (0) pristine 0.5 ML Gr on Ir(111), (1) after exposure to 200 L O_2 at 450 K, and (2) after subsequent exposure to 100 L H_2 at 300 K.

Figure S1 is identical to figure 1(b) of the main paper, except for the third column, which represents Ir 4f core level spectra. In step (0) with Ir(111) covered by 0.5 ML Gr the Ir 4f spectrum is characterized by the bulk component Ir_B (grey) and the surface component Ir_0 (orange). Since Gr does not interact chemically with the Ir(111) surface, the spectrum is indistinguishable from a spectrum of bare Ir(111). After O_2 exposure in step (1) the Ir_0 surface component has vanished and two new components Ir_1 (light red) and Ir_2 (dark red) appear that correspond to Ir surface atoms bound to 1 or 2 chemisorbed oxygen atoms, respectively. After additional H_2 exposure the initial Ir 4f spectrum is largely recovered. Specifically, the re-appearance of the Ir_0 surface component close to the original intensity indicates the absence of chemisorption to the surface. This implies the absence of chemisorbed oxygen (except for very small residual amount oxygen giving rise to the tiny remaining Ir_1 component).

In situ **exposure to O_2 and H_2**

0.5 ML Gr intercalated by a p(2×1)-O structure (corresponding to step (1) in figure 1(b) of the main article) was first exposed to $1{\cdot}10^{-7}$ mbar of H_2 and subsequently to $1{\cdot}10^{-7}$ mbar of O_2 at room temperature. During the gas exposures the O 1s and C 1s regions were monitored simultaneously, without moving the sample. A photon energy of 625 eV was used for both core levels. Figure S2(a) and (b) show the evolution of the O 1s region and C 1s region, respectively. The filled vertical lines indicate the times where the H_2 and O_2 exposures began, and the dashed vertical lines indicate the end of the respective gas exposure. The horizontal lines indicate the positions of the fitted peaks in figure 1(b) of the main article. The lower horizontal axes display the dose of the respective gas, while the upper axes show the time passed since the first spectrum. Panels (c) and (d) shows the areas of the fitted components. A linear background was subtracted prior to fitting. The components used to fit the spectra are the same as described in the main article, except for a larger GFWHM of the C 1s spectra due to the resolution at the photon energy used here. The peak areas are normalized to the average total area of the spectra before gas exposure (*i.e.* the average of the first five spectra). The precise local coverage of the mixed OH-H_2O phase causing the C_2 component can be found from the absolute intensities of the O_{OH} and O_{H_2O} components (measured as precise as possible without moving the sample or the photon energy) and the relative intensity of the C_2 component.

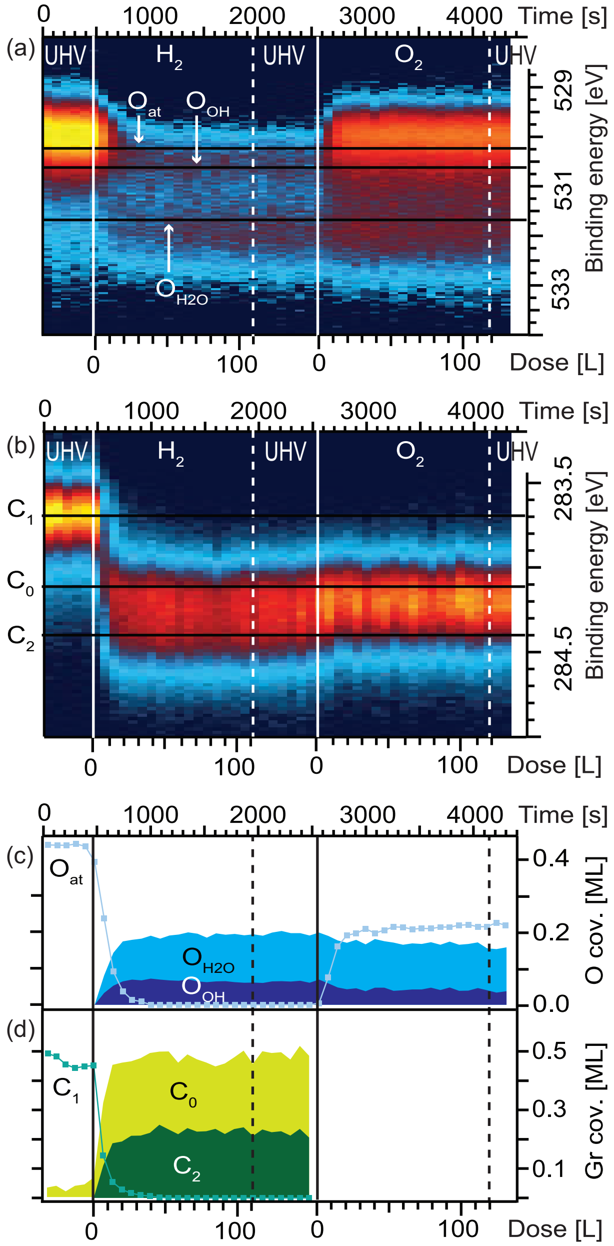

Figure S2: Evolution of the O 1s (a) and C 1s (b) regions measured during exposure to $1 \cdot 10^{-7}$ mbar of H_2 and subsequently $1 \cdot 10^{-7}$ mbar of O_2. Initially (*i.e.* time = 0 s) the sample consists of 0.5 ML graphene/Ir(111) intercalated by a p(2×1)-O structure, corresponding to step (1) in figure 1(b) of the main article. The intensity of the fitted O 1s and C 1s components are shown in panel (c) and (d), respectively.

Temperature programmed desorption

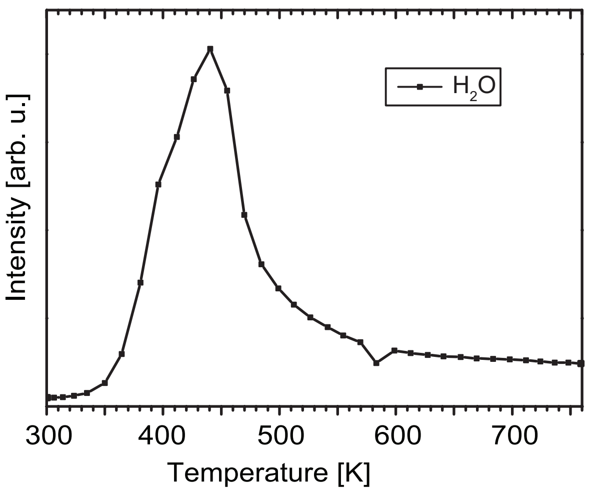

Figure S3: TPD spectrum of H_2O desorption from 0.8 ML Gr after 4 cycles of O_2 and H_2 exposure. A temperature ramp of 5 K/s was used. No background correction was used. TPD data were recorded with a quadrupole mass spectrometer equipped with a Feulner cup.[1]

TPD spectrum recorded after sequentially dosing 4 cycles of 750 L O_2 and 100 L H_2. The first O_2 exposure was done at 520 K to ensure oxygen intercalation. All subsequent H_2 and O_2 exposures were done at room temperature. One single H_2O desorption peak is observed at 440 K significantly above the water desorption peak temperature for pure water (170 K)[2–4] or a mixed OH/H_2O phase (210-235 K)[2,3] on Ir(111) without Gr.

STM movie

STM movie showing three O-intercalated Gr flakes during H_2 exposure at $5{\cdot}10^{-9}$ mbar. The start of the H_2-dosing is marked as a black frame in the movie. The total H_2 dose is about 7.2 L. The acquisition time is 85 seconds per image. The image size is 71 nm $\times$ 71 nm. All images were recorded at room temperature with a tunneling current of 5.5 nA, and a bias voltage of -1V.

Cycles of H_2 and O_2 exposure

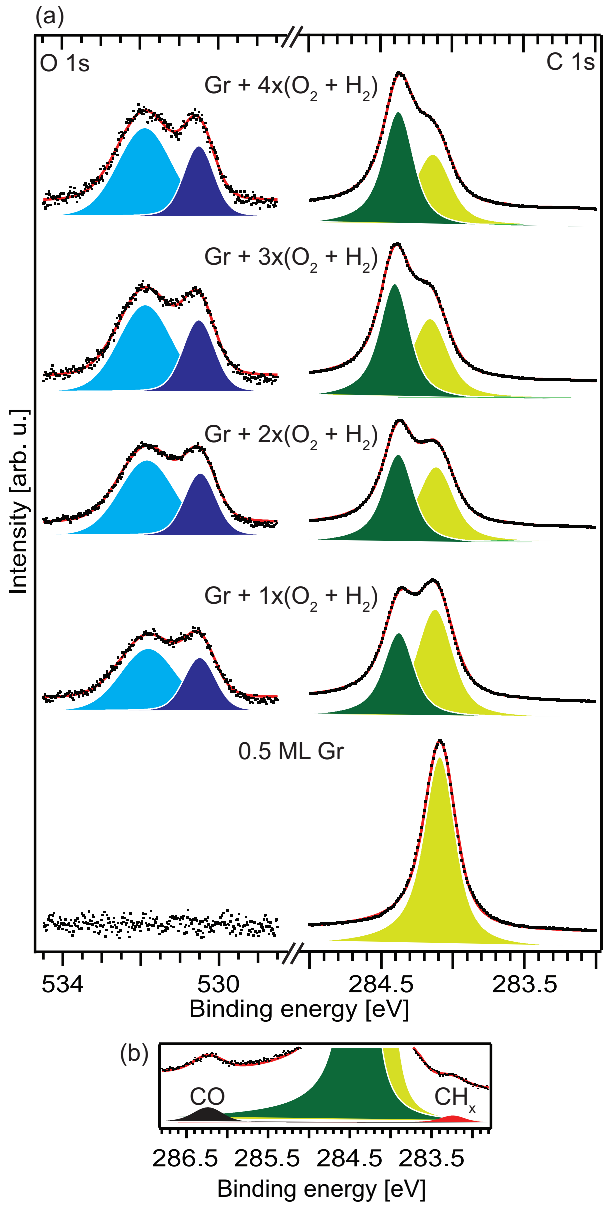

Figure S4: (a) O 1s (left) and C 1s (right) spectra corresponding to pristine 0.5 ML Gr (bottom), and after exposure to an additional cycle of 200 L O_2 and 100 L H_2 for each pair of O 1s and C 1s spectra. (b) The same spectrum as the top one in (a), with an exteneded binding energy region and adjusted intensity scale to show the low intensity peaks.

The O 1s and C 1s spectra of 0.5 ML graphene exposed to cycles of O_2 and H_2 are shown in figure S4(a). Lowest spectra: pristine 0.5 ML Gr. Second spectra: after exposure to 200 L O_2 at 450 K and 100 L H_2 at 300 K. Thereafter each spectra has been exposed to an

additional cycle of 200 L O_2 and 100 L H_2 at 300 K. The lowest two are also shown in figure 1 in the main article, the third from the bottom is shown in figure 4(a). The areas in figure 4(c) are calculated from the spectra shown here.

Also present in the C 1s spectra after H_2 exposure are a component corresponding to adsorbed CO, located at 286.2 eV, and a carbon component located at 283.3 eV, as can be seen in figure S4(b). The CO adsorb from the background and the component increase with time. The area of the CO component relative to the total graphene area is always less than 0.5 %. The component at 283.2 eV we tentatively assign to a CH specie adsorbed on Ir(111),[5] forming either through reactions between the H_2 and CO on the surface or through a beam induced reaction. The area of this carbon component is always less than 1 % of the total graphene area.

O_6H_2 phase

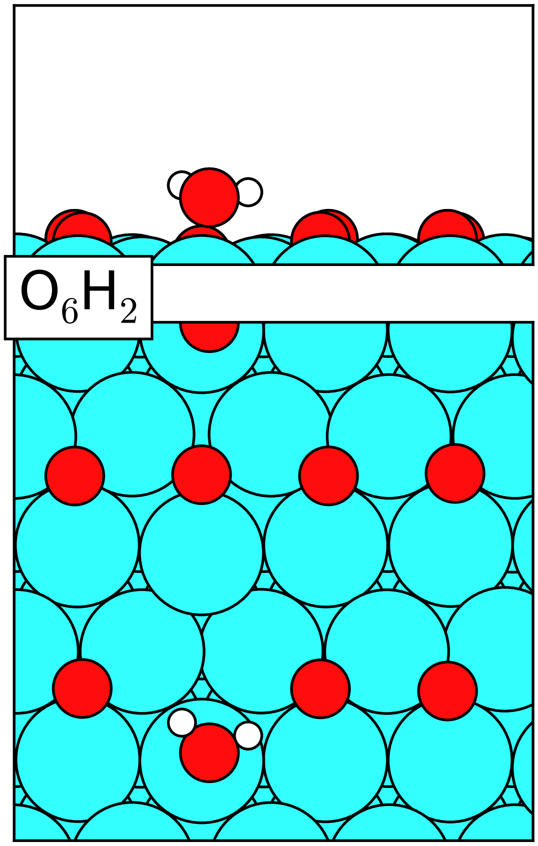

Figure S5: The most stable structure of the H-poor O_6H_2 phase found with DFT

References

(1) Feulner, P. & Menzel, D. Simple ways to improve flash desorption measurements from single-crystal surfaces. *J. Vac. Sci. Technol.* **17,** 662-663 (1980)

(2) Shavorskiy, A., Gladys, M. J. & Held, G. Chemical composition and reactivity of water on hexagonal Pt-group metal surfaces. *Phys. Chem. Chem. Phys.* **10,** 6150-6159 (2008)

(3) Pan, M., Hoang, S. & Mullins, C. B. Interaction of water with the clean and oxygen pre-covered Ir(111) surface. *Catal. Today* **160,** 198-203 (2011)

(4) Standop, S. Water adsorption and ion induced defect formation: A comparative study of graphene and noble metal surfaces. *Ph.D Thesis, Universität zu Köln* (2013)

(5) Weststrate, C. J., Ludwig, W., Bakker, J. W., Gluhoi, A. C. & Nieuwenhuys, B. E. Ethanol adsorption, decomposition and oxidation on Ir(111): a high resolution XPS study. *ChemPhysChem* **8,** 932-937 (2007)
